# Analysis of bacteria associated with honeys of different geographical and botanical origin using two different identification approaches: MALDI-TOF MS and 16S rDNA PCR technique

**DOI:** 10.1371/journal.pone.0217078

**Published:** 2019-05-23

**Authors:** Paweł Pomastowski, Michał Złoch, Agnieszka Rodzik, Magda Ligor, Markus Kostrzewa, Bogusław Buszewski

**Affiliations:** 1 Interdisciplinary Center for Modern Technologies, Nicolaus Copernicus University in Torun, Torun, Poland; 2 Department of Environmental Chemistry and Bioanalytics, Faculty of Chemistry, Nicolaus Copernicus University in Torun, Torun, Poland; 3 Bruker Daltonik GmbH, Bremen, Germany; University of San Agustin, PHILIPPINES

## Abstract

In the presented work identification of microorganisms isolated from various types of honeys was performed. Martix-assisted laser desorption/ionization time-of-flight mass spectrometry (MALDI-TOF MS) and 16S rDNA sequencing were applied to study environmental bacteria strains.With both approches, problematic spore-forming *Bacillus spp*, but also *Staphylococcus spp*., *Lysinibacillus spp*., *Micrococcus spp*. and *Brevibacillus spp* were identified. However, application of spectrometric technique allows for an unambiguous distinction between species/species groups e.g.*B*. *subtilis* or *B*. *cereus* groups. MALDI TOF MS and 16S rDNA sequencing allow for construction of phyloproteomic and phylogenetic trees of identified bacterial species. Furthermore, the correlation beetween physicochemical properties, geographical and botanical origin and the presence bacterial species in honey samples were investigated.

## Introduction

Honey, a supersaturated solution of sugars (mostly glucose and fructose) produced by *Apis mellifera*, is the first and most reliable sweetener used by human beings [1]. In addition to the high nutritional value that makes it a highly consumed food product around the world, honey is also known for its healing, antioxidant as well as antimicrobial properties [2],[3]. High antibacterial effect of honey is primarily related to the high sugar concentration. This implicates its hyperosmotic nature, high viscosity, and low water content which in consequence prevent the growth or even survival of most vegetative forms of human pathogenic microorganisms by their desiccation as well as limited atmospheric oxygen penetration [4],[5]. Moreover, natural acidity of this product, the ability to produced hydrogen peroxide, and presence of numerous phytochemical factors such as phenols, terpenes or flavonoids (e.g. pinacombrin) cause that honey has a permanent place in the treatment of wound infections and burns [6],[7],[8].

Despite its richness in sugar and inhibins, honey cannot be considered as sterile since many studies proved that it is subject to bacterial and fungal contaminations derived from two kind of sources: primary and secondary [9],[10],[11]. The first ones include pollen, dirt, dust, air, water, flowers, as well as the digestive tracts of honeybees and are considered as natural sources which are difficult to control [12] [13]. The secondary sources are those arising from the honey manipulation by people, thus are closely connected with hygiene of processing, handling, and storage [11]. Such sources includes skin, mouth, and nose of food handlers, equipment, buildings as well as cross-contamination during harvest and processing in honey houses, however, they can be easy control by the application of good manufacturing practices [14],[15]. Microorganisms found in honey must demonstrate the ability to withstand the concentrated sugar, acidity and other severe conditions, thus in most cases they are present in latent forms (dormant) such as spores [4]. Therefore, besides different species of molds and yeasts, the major microbiological contaminants of honey include spore forming bacteria,e.g. *Clostridium* spp. and *Bacillus* spp. [16], [17], [13]. Although studies on microbial contamintation of honey are mainly focused on the occurrence of *C*. *botulinum* [18], *Bacillus* spp. are also microorganisms of concern since some of them (e.g. *B*. *cereus*) are associated with spoilage of food and foodborne outbreaks [15], [19].

In contrast to the physicochemical properties, microbial contamination of honey has not been thoroughly investigated so far, which is reflected in the lack of proper legislation concerning this issue in the European Union [13]. In the available literature related to honey microbiota investigation, the technique used for microorganisms identification most frequently still is 16S rDNA sequencing, also considered as a gold standard of microorganism identification. However, molecular assays require a high level of expertise and can be quite expensive. Thus, they are not ideally suitable for routine identification which primarily requires rapidity and low cost at the same time [20]. Therefore, since last 10 years a novel identification approach called Matrix-assisted laser desorption/ionization time-of-flight mass spectrometry (MALDI-TOF MS) steadily is gaining in popularity due to its high accuracy of identification, the robustness as well as rapidity of obtaining results [21]. This technique relies mostly on the detection of microbial protein patterns (proteomic approach), and analysis of such large biomolecules is possible thanks to the so-called soft ionization mechanism [22], [23]. To date, there are no scientific reports on the wider use of MALDI-TOF MS in the investigation of the microbiological composition of different types of honey. Therefore, the main goal of this study was to identify bacterial species present in honey samples using two different diagnostic approaches–genomic (16S rDNA sequencing) and proteomic (MALDI-TOF MS via MALDI Biotyper platform) in order to compare their usefulness in the characterization of the bacterial composition and thus in controlling microbiological purity of honey. Moreover, the influence of the physicochemical features and geographical origin of honey on their bacterial composition was analyzed.

## Results

### Physicochemical properties of honey

Investigated honey samples significantly differed in both pH, total acidity, color, and electrical conductivity (Table 1). pH ranged from 3.3 to 5.0, however, most of the honeys (70%) had a pH value ≤4.0. The highest pH values were noted for both honeydew honeys and goldenrod nectar—>4.3. Considering acidity, values of TA ranged from 12.8 (*RNSK*) to 44.0 (*BBS*) and in most cases (70%) not exceeding 30 mval/kg. Investigated honeys demonstrated high variety in their color–from white (18–34 mm in Pfund scale) to dark amber (>118 mm) and were mostly represented by darker ones– 65%. EC ranged from 0.183–0.187 for rape nectar to 1.236–1.241 for honeydew honeys, however, most of the samples were characterized by lower conductivity (<0.500).

**Table 1:** List of investigated honeys with physicochemical parameters.

Country	Name	Acronym	pH	Total acidity [mval/ kg]	Color [mm]	Electrical conductivity [mS/cm]
**Poland**	Buckwheat *Sądecki Bartnik*	*BSB*	3.9	35.0	107.50	0.310
	buckwheat *Barć Świętokrzyska*	*BBS*	3.6	44.0	184.63	0.398
	buckwheat *Karczowiska Górne*	*BKG*	4.0	29.0	231.05	0.467
	multiflorous *Sądecki Bartnik*	*MSB*	3.6	16.0	75.32	0.292
	multiflorous *Wilga*	*MW*	4.3	30.0	59.72	0.854
	multiflorous *B Szymanski*	*MBS*	3.7	16.8	18.25	0.249
	honeydew *Sądecki Bartnik*	*HSB*	4.5	31.0	151.08	1.236
	honeydew *Sądecki Bartnik Stróże*	*HSBS*	4.4	35.0	108.99	1.241
	rape *Sądecki Bartnik*	*RSB*	3.6	15.0	114.07	0.183
	goldenrod *Słoneczna Pasieka Stryków*	*GSPS*	3.3	42.7	58.85	0.331
	goldenrod nectar	*GN*	5.0	17.0	243.93	0.416
	rape nectar *Solec Kujawski*	*RNSK*	4.0	12.8	34.34	0.187
	sunflower Olekszyn	*SO*	3.9	21.0	62.07	0.404
	lime *Tomasz Strecker Apiary Łysomice*	*LTSAL*	3.9	34.0	281.56	0.537
**Australia**	bush *Tasmania*	*BT*	4.2	26.0	129.66	0.677
	leatherwood *Tasmania*	*LT*	4.3	21.0	88.81	0.704
	clover *Tasmania*	*CT*	3.6	22.0	20.10	0.255
**Italy**	multiflorous *Miele di Millefiori*	*MMDM*	3.8	26.0	106.39	0.410
**Ukraine**	sunflower *Black Sea Bartnik*	*SSB*	3.7	25.0	114.44	0.374
**Portugal**	forest *Madeira*	*FM*	4.0	30.0	150.96	0.670

Pfund scale: <9 –water white; 9–17 –extra white; 18–34 –white; 35–50 –extra light amber; 51–85 –light amber; 86–114 –amber; >114 –dark amber

### Bacteria isolation

As a result of the isolation, 38 bacterial colonies were selected for further identification (H1 –H38). From most of the investigated honeys (15) one or two colonies were chosen due to very low bacterial content, while 5 samples–*BSB*, *MW*, *GSPS*, *SO*, and *MMDM* were represent by 3 to 5 colonies proportionally to bacterial abundance.

### 16S rDNA identification

**Fig 1:**
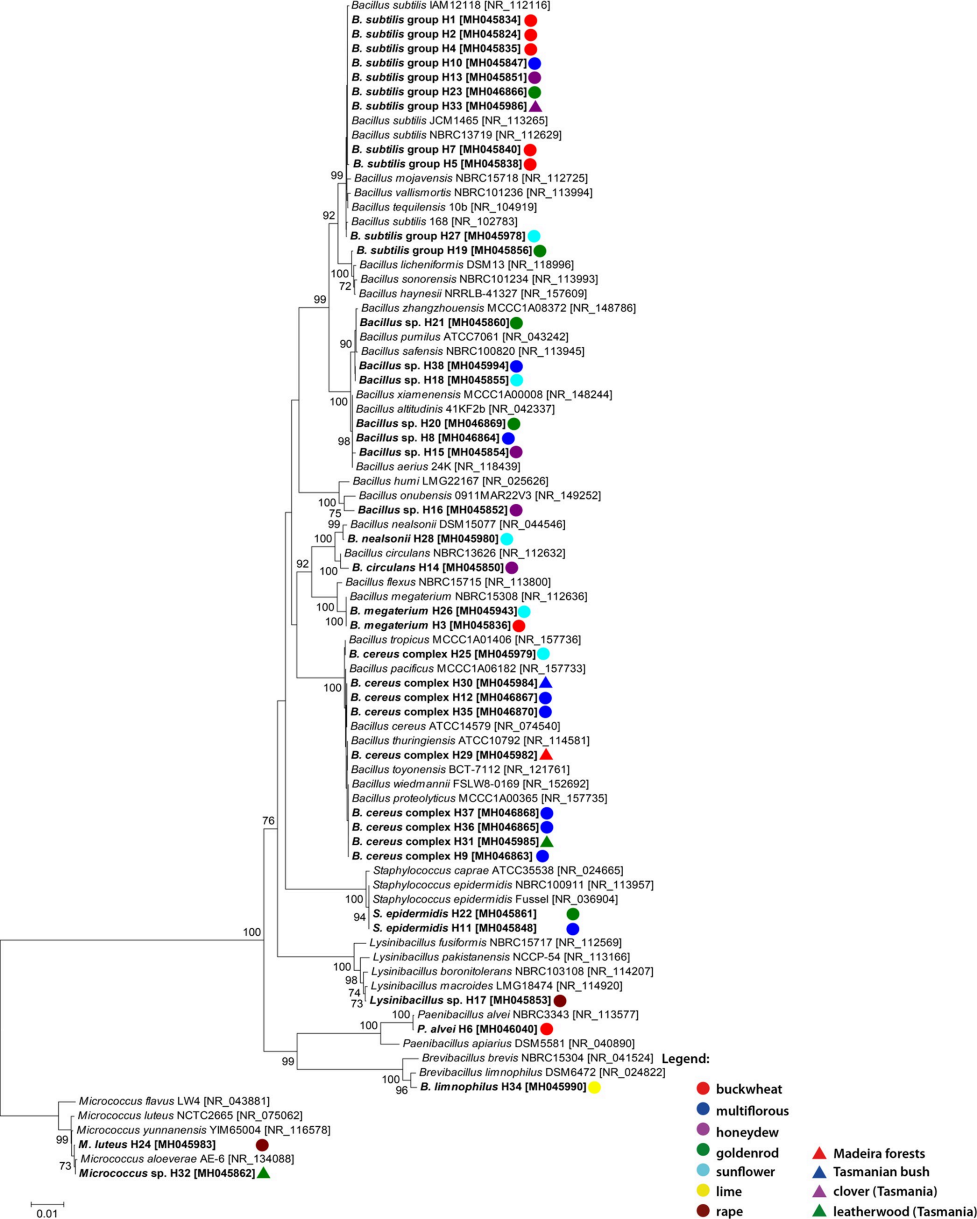
The phylogenetic tree of identified bacterial species based on the 16S rDNA analysis. The phylogenetic analysis performed including the bootstrap value. Botanical origin was marked with different colors.

Based on the sequencing of the 16S rDNA region, all isolated bacterial strains were identified and represent group of Gram-positive bacteria (Fig 1). Almost 95% of isolates belonged to the class *Bacilli* (phylum *Firmicutes*) among which 34 strains were able to produce endospores—mostly represent by *Bacillaceae* family (~89%)–while 2 strains were identified as *Staphylococcus epidermidis* were characterized by both nonmotility and nonsporulation. Within *Bacillus* genus, *B*. *subtilis* group was the most frequently identified species (11 out of 31 strains) followed by *B*. *cereus* complex (9). However, the reliable species distinction within the mentioned groups was impossible due to obtained small differences in 16S rDNA sequences (< 0.5%) (Table 2). Similar phenomenon was observed for isolates most similar to members of *B*. *pumilus* group (H18, H21, and H38) and its closely related relatives such as *B*. *altitudinis*, *B*. *xiamensis* or *B*. *aerius*–H8, H15, H20. Moreover, individual cases revealed the presence of *B*. *megaterium* (2 cases), *B*. *circulans* as well as *B*. *nealsonii*. Regarding other genera within *Firmicutes* phylum, two *Paenibacillaceae* strains (*P*. *alvei* and *Brevibacillus limnophilus*) and one *Lysinibacillus* sp. were detected. Only two isolates belonged to another phylum of bacteria–H24 isolated from rape nectar (*RNSK*) and H32 from leatherwood honey (*LT*)–both classified as *Micrococcus* genus members (phylum *Actinobacteria*). Obtained phylogenetic tree showed grouping of isolates according to individual species as well as revealed presence of bigger clusters containing closely related species– 1. *Bacillus subtilis* group, 2. *Bacillus cereus* group, as well as 3. *Bacillus pumilus* group and its close relatives (Fig 1). Nevertheless, considering level of identification, only for 24% isolates obtained reliable identification to the species while in 76% of cases observed classification at the genus level.

**Table 2:** The result of bacteria identification based on 16S rDNA sequencing.

Strain	Related species from NCBI [Accesion number]	Identity [%]	Given accesion number
H1.	*Bacillus subtilis* JCM1465 [NR_113265] *Bacillus tequilensis* 10b [NR_104919] *Bacillus mojavensis* NBRC15718 [NR_112725]	99.9 99.9 99.7	MH045834
H2.	*Bacillus subtilis* JCM1465 [NR_113265] *Bacillus tequilensis* 10b [NR_104919] *Bacillus mojavensis* NBRC15718 [NR_112725]	99.9 99.9 99.7	MH045824
H3.	*Bacillus megaterium* NBRC15308 [NR_112636] *Bacillus flexus* NBRC15715 [NR_113800]	100 98.9	MH045836
H4.	*Bacillus subtilis* JCM1465 [NR_113265] *Bacillus tequilensis* 10b [NR_104919] *Bacillus mojavensis* NBRC15718 [NR_112725]	99.9 99.9 99.7	MH045835
H5.	*Bacillus subtilis* JCM1465 [NR_113265] *Bacillus tequilensis* 10b [NR_104919] *Bacillus vallismortis* NBRC101236 [NR_104919]	99.9 99.8 99.6	MH045838
H6.	*Paenibacillus alvei* NBRC3343 [NR_113577] *Paenibacillus apiarius* DSM5581 [NR_040890]	99.7 96.9	MH046040
H7.	*Bacillus subtilis* JCM1465 [NR_113265] *Bacillus subtilis* NBRC13719 [NR_112629] *Bacillus tequilensis* 10b [NR_104919]	100 100 99.7	MH045840
H8.	*Bacillus altitudinis* 41KF2b [NR_042337] *Bacillus aerius* 24K [NR_118439] *Bacillus xiamenensis* MCCC1A00008 [NR_148244]	100 100 99.9	MH046864
H9.	*Bacillus wiedmannii* strain FSL W8-0169 [NR_152692] *Bacillus proteolyticus strain* MCCC1A00365 [NR_157735] *Bacillus cereus* ATCC14579 [NR_074540]	100 100 99.9	MH046863
H10.	*Bacillus subtilis* JCM1465 [NR_113265] *Bacillus tequilensis* 10b [NR_104919] *Bacillus mojavensis* NBRC15718 [NR_112725]	99.9 99.9 99.7	MH045847
H11.	*Staphylococcus epidermidis* Fussel [NR_036904] *Staphylococcus caprae* ATCC35538 [NR_024665]	99.9 99.4	MH045848
H12.	*Bacillus cereus* ATCC14579 [NR_074540] *Bacillus tropicus* MCCC1A01406 [NR_157736] *Bacillus proteolyticus strain* MCCC1A00365 [NR_157735]	100 99.9 99.9	MH046867
H13.	*Bacillus subtilis* JCM1465 [NR_113265] *Bacillus tequilensis* 10b [NR_104919] *Bacillus mojavensis* NBRC15718 [NR_112725]	99.9 99.9 99.7	MH045851
H14.	*Bacillus circulans* NBRC13626 [NR_112632] *Bacillus nealsonii* DSM15077 [NR_044546]	99.7 98.1	MH045850
H15.	*Bacillus altitudinis* 41KF2b [NR_042337] *Bacillus aerius* 24K [NR_118439] *Bacillus xiamenensis* MCCC1A00008 [NR_148244]	99.9 99.9 99.8	MH045854
H16.	*Bacillus onubensis* 0911MAR22V3 [NR_149252] *Bacillus humi* LMG22167 [NR_025626]	98.8 98.7	MH045852
H17.	*Lysinibacillus macroides* LMG18474 [NR_114920] *Lysinibacillus boronitolerans* NBRC103108 [NR_114207] *Lysinibacillus pakistanensis* NCCP-54 [NR_113166]	99.7 99.4 99.3	MH045853
H18.	*Bacillus pumilus* ATCC7061 [NR_043242] *Bacillus zhangzhouensis* MCCC1A08372 [NR_148786] *Bacillus safensis* NBRC100820 [NR_113945]	99.9 99.9 99.8	MH045855
H19.	*Bacillus haynesii* NRRL B-41327 [NR_157609] *Bacillus licheniformis* DSM13 [NR_118996] *Bacillus sonorensis* NBRC 101234 [NR_113993]	99.7 99.6 99.5	MH045856
H20.	*Bacillus altitudinis* 41KF2b [NR_042337] *Bacillus aerius* 24K [NR_118439] *Bacillus xiamenensis* MCCC1A00008 [NR_148244]	100 100 99.9	MH046869
H21.	*Bacillus pumilus* ATCC7061 [NR_043242] *Bacillus safensis* NBRC100820 [NR_113945]	99.9 99.8	MH045860
H22.	*Staphylococcus epidermidis* Fussel [NR_036904] *Staphylococcus epidermidis* NBRC100911 [NR_113957] *Staphylococcus caprae* ATCC35538 [NR_024665]	100 99.9 99.5	MH045861
H23.	*Bacillus subtilis* JCM1465 [NR_113265] *Bacillus subtilis* IAM12118 [NR_112116] *Bacillus tequilensis* 10b [NR_104919]	100 99.9 99.9	MH046866
H24.	*Micrococcus luteus* NCTC2665 [NR_075062] *Micrococcus flavus* LW4 [NR_043881	99.6 98.3	MH045862
H25.	*Bacillus tropicus* MCCC1A01406 [NR_157736] *Bacillus cereus* ATCC14579 [NR_074540] *Bacillus wiedmannii* strain FSL W8-0169 [NR_152692]	99.9 99.9 99.9	MH045979
H26.	*Bacillus megaterium* NBRC15308 [NR_112636] *Bacillus flexus* NBRC15715 [NR_113800]	100 98.9	MH045943
H27.	*Bacillus tequilensis* 10b [NR_104919] *Bacillus subtilis* 168 [NR_102783] *Bacillus mojavensis* NBRC15718 [NR_112725]	99.9 99.9 99.7	MH045978
H28.	*Bacillus nealsonii* DSM15077 [NR_044546] *Bacillus circulans* NBRC13626 [NR_112632]	99.4 98.9	MH045980
H29.	*Bacillus thuringiensis* ATCC10792 [NR_114581] *Bacillus toyonensis* BCT-7112 [NR_121761] *Bacillus pacificus* MCCC1A06182 [NR_157733]	100 100 99.9	MH045982
H30.	*Bacillus cereus* ATCC14579 [NR_074540] *Bacillus tropicus* MCCC1A01406 [NR_157736] *Bacillus wiedmannii* strain FSL W8-0169 [NR_152692]	100 99.9 99.9	MH045984
H31.	*Bacillus wiedmannii* strain FSL W8-0169 [NR_152692] *Bacillus proteolyticus strain* MCCC1A00365 [NR_157735] *Bacillus cereus* ATCC14579 [NR_074540]	99.9 99.9 99.9	MH045985
H32.	*Micrococcus aloeverae* AE-6 [NR_134088] *Micrococcus yunnanensis* YIM65004 [NR_116578] *Micrococcus luteus* NCTC2665 [NR_075062]	99.9 99.8 99.5	MH045983
H33.	*Bacillus tequilensis* 10b [NR_104919] *Bacillus subtilis* JCM1465 [NR_113265] *Bacillus mojavensis* NBRC15718 [NR_112725]	99.9 99.9 99.9	MH045986
H34.	*Brevibacillus limnophilus* DSM6472 [NR_024822] *Brevibacillus brevis* NBRC15304 [NR_041524]	99.2 98.6	MH045990
H35.	*Bacillus cereus* ATCC14579 [NR_074540] *Bacillus tropicus* MCCC1A01406 [NR_157736] *Bacillus proteolyticus strain* MCCC1A00365 [NR_157735]	100 99.9 99.9	MH046870
H36.	*Bacillus wiedmannii* strain FSL W8-0169 [NR_152692] *Bacillus proteolyticus strain* MCCC1A00365 [NR_157735] *Bacillus cereus* ATCC14579 [NR_074540]	100 100 99.9	MH046865
H37.	*Bacillus wiedmannii* strain FSL W8-0169 [NR_152692] *Bacillus proteolyticus strain* MCCC1A00365 [NR_157735] *Bacillus cereus* ATCC14579 [NR_074540]	100 100 99.9	MH046868
H38.	*Bacillus pumilus* ATCC7061 [NR_043242] *Bacillus zhangzhouensis* MCCC1A08372 [NR_148786] *Bacillus safensis* FO-36b [NR_041794]	99.9 99.8 99.7	MH045994

### MALDI-TOF/MS identification

**Fig 2:**
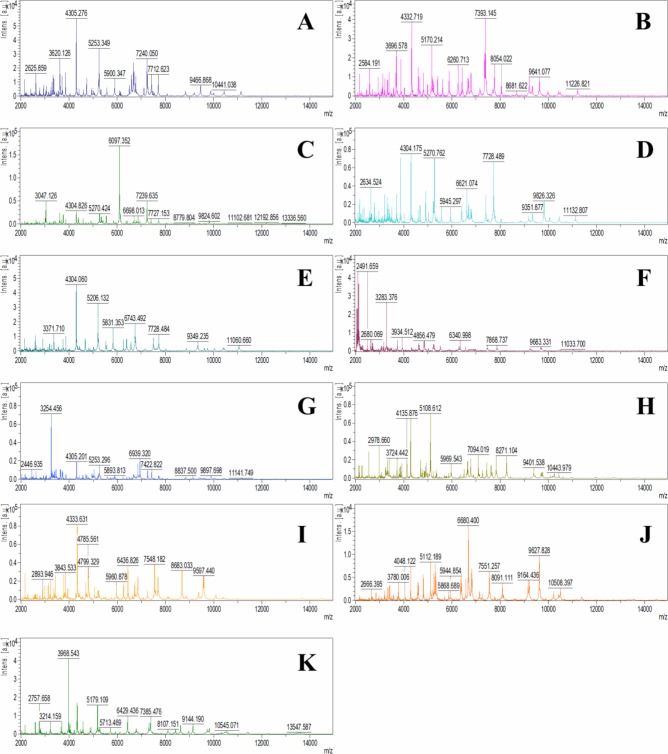
Exemplary MS spectra obtained for bacterial strains identified as different species. A–H1 (*B*. *subtilis*); B–H9 (*B*. *cereus*); C–H8 (*B*. *pumilus*); D–H20 (*B*. *altitudinis*); E–H3 (*B*. *megaterium*); F–H14 (*B*. *circulans*); G–H19 (*B*. *licheniformis*); H–H6 (*P*. *alvei*); I–H17 (*L*. *boronitolerans*); J–H11 (*S*. *epidermidis*); K–H24 (*M*. *luteus*).

Used procedure for sample preparation and MALDI-TOF MS analysis allowed to obtain MS spectra for all tested bacterial strains (Fig 2). 36 and 35 bacterial strains were classified in single spectrum and MSP mode using MALDI Biotyper platform, respectively (Table 3). Taking into account raw spectra, 69% isolates were classified to the species level as high-confidence identification (Score value >1.999) from which 42% (29% in total) were identified with a very high log(score) (>2.3), mostly related to *B*. *cereus*. Moreover, score values for all isolates identified as *B*. *cereus* were > 1.999 (high confidence level), while in case of *B*. *subtilis* 36% isolates were identified only at the low confidence level (1.700–1.999). In total, a quarter of the identifications have reached only the low confidence level and in the case of 2 strains–H28 (*SO*) and H34 (*LTSAL*)–no reliable identification was obtained. Considering consistency of obtained results, half of the identifications reached species level, 42% genus level, while 8% was defined as neither species nor genus consistency. In MSP mode, percentage of identification at the high confidence level was slightly higher compared to the raw spectra– 74%, however, number of not reliable identification raised from 2 to 3 strains–in addition to H28 and H34 isolates also H19 strain failed (*GSPS*). Similar to the raw spectra mode, all *B*. *cereus* strains were identified at high confidence level (Table 3). while 2 strains of *B*. *subtilis* were recognized only at low confidence level. Consistency of identification in MSP mode was also higher than in the case of raw spectra analysis– 61% samples with species consistency. Omitting not reliable identifications, results of organisms matching for each isolate were similar except for bacteria H17 derived from *RSB* honey, which was identified as *L*. *boronitolerans* based on the raw spectra analysis while in MSP mode as *L*. *fusiformis*–both on high confidence level.

**Table 3:** The result of bacteria identification via MALDI Biotyper platform based on raw spectra (RAW) and mainspectrum (MSP).

	RAW			MSP		
St.	Best match	Score value	Rank	Best match	Score value	Rank
H1	***B*. *subtilis***	**2.02**	**++(A)**	***B*. *subtilis***	**2.06**	**++(A)**
H2	***B*. *subtilis***	**2.37**	**+++(A)**	***B*. *subtilis***	**2.21**	**++(A)**
H3	***B*. *megaterium***	**2.44**	**+++(A)**	***B*. *megaterium***	**2.44**	**+++(A)**
H4	***B*. *subtilis***	**1.96**	**+(B)**	***B*. *subtilis***	**1.93**	**+(B)**
H5	***B*. *subtilis***	**2.43**	**+++(B)**	***B*. *subtilis***	**2.36**	**+++(B)**
H6	***P*. *alvei***	**2.15**	**++(A)**	***P*. *alvei***	**2.24**	**++(A)**
H7	***B*. *subtilis***	**2.10**	**++(A)**	***B*. *subtilis***	**2.03**	**++(A)**
H8	***B*. *pumilus***	**1.91**	**+(B)**	***B*. *pumilus***	**1.89**	**+(B)**
H9	***B*. *cereus***	**2.30**	**+++(A)**	***B*. *cereus***	**2.27**	**++(A)**
H10	***B*. *subtilis***	**2.11**	**++(A)**	***B*. *subtilis***	**2.06**	**++(A)**
H11	***S*. *epidermidis***	**2.10**	**++(A)**	***S*. *epidermidis***	**2.11**	**++(A)**
H12	***B*. *cereus***	**2.23**	**++(A)**	***B*. *cereus***	**2.23**	**++(A)**
H13	***B*. *subtilis***	**2.29**	**++(A)**	***B*. *subtilis***	**2.23**	**++(A)**
H14	***B*. *circulans***	**2.18**	**++(A)**	***B*. *circulans***	**2.19**	**++(A)**
H15	***B*. *pumilus***	**1.85**	**+(B)**	***B*. *pumilus***	**1.75**	**+(B)**
H16	***B*. *subtilis***	**1.83**	**+(B)**	***B*. *subtilis***	**1.92**	**+(B)**
H17	***L*. *boronitolerans***	**2.16**	**++(C)**	***L*. *fusiformis***	**2.09**	**++(B)**
H18	***B*. *pumilus***	**1.79**	**+(B)**	***B*. *pumilus***	**1.86**	**+(B)**
H19	***B*. *licheniformis***	**1.87**	**+(B)**	***-***	**1.62**	**-(C)**
H20	***B*. *altitudinis***	**1.79**	**+(B)**	***B*. *altitudinis***	**1.92**	**+(B)**
H21	***B*. *pumilus***	**2.03**	**++(A)**	***B*. *pumilus***	**2.07**	**++(A)**
H22	***S*. *epidermidis***	**2.24**	**++(A)**	***S*. *epidermidis***	**2.27**	**++(A)**
H23	***B*. *subtilis***	**2.38**	**+++(A)**	***B*. *subtilis***	**2.34**	**+++(A)**
H24	***M*. *luteus***	**2.21**	**++(A)**	***M*. *luteus***	**2.25**	**++(A)**
H25	***B*. *cereus***	**2.25**	**++(A)**	***B*. *cereus***	**2.31**	**+++(B)**
H26	***B*. *cereus***	**2.34**	**+++(B)**	***B*. *cereus***	**2.30**	**+++(A)**
H27	***B*. *subtilis***	**1.96**	**+(B)**	***B*. *subtilis***	**2.01**	**++(A)**
H28	***-***	**1.63**	**-(C)**	***-***	**1.26**	**-(C)**
H29	***B*. *mycoides***	**2.31**	**+++(B)**	***B*. *mycoides***	**2.21**	**++(A)**
H30	***B*. *cereus***	**2.37**	**+++(A)**	***B*. *cereus***	**2.37**	**+++(B)**
H31	***B*. *cereus***	**2.27**	**++(B)**	***B*. *cereus***	**2.25**	**++(A)**
H32	***M*. *luteus***	**2.23**	**++(A)**	***M*. *luteus***	**2.35**	**+++(A)**
H33	***B*. *subtilis***	**1.99**	**+(B)**	***B*. *subtilis***	**2.03**	**++(A)**
H34	***-***	**1.40**	**-(C)**	***-***	**1.33**	**-(C)**
H35	***B*. *cereus***	**2.42**	**+++(B)**	***B*. *cereus***	**2.39**	**+++(B)**
H36	***B*. *cereus***	**2.37**	**+++(B)**	***B*. *cereus***	**2.38**	**+++(B)**
H37	***B*. *cereus***	**2.31**	**+++(A)**	***B*. *cereus***	**2.26**	**++(A)**
H38	***B*. *pumilus***	**1.89**	**+(B)**	***B pumilus***	**1.76**	**+(A)**

The level of identification: +++—highly probable species identification (2.300–3.000); ++—secure genus identification, probable species identification (2.000–2.299); +—probable genus identification (1.700–1.999);—not reliable identification (0.000–1.699).

Consistency status: A–high (species); B–low (genus); C–none.

**Fig 3:**
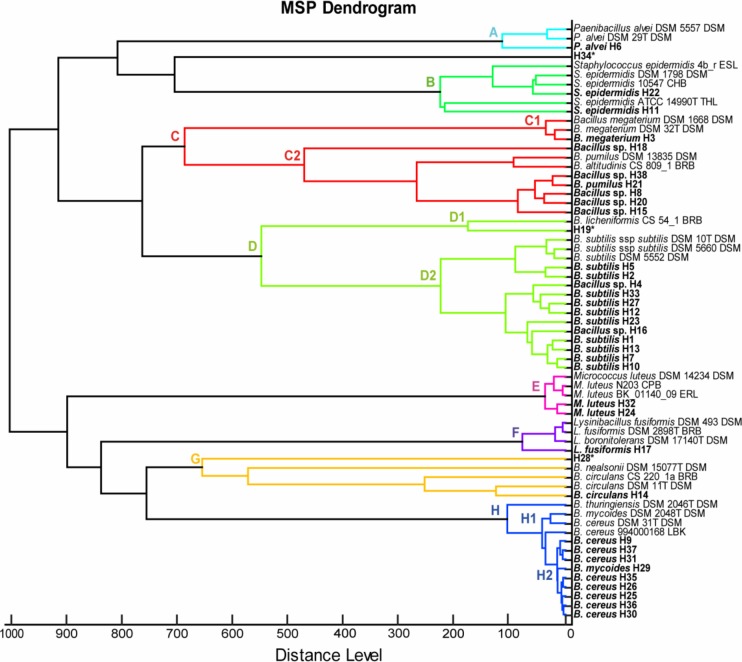
Phyloproteomic tree of investigated bacterial strains based on the MSP identification via MALDI Biotyper platform.

Phyloproteomic relationships between isolates presented on MSP dendrogram (Fig 3) revealed the presence of 8 groups of closely related bacterial species (A–H). Similar to the phylogenetic tree, 3 bigger clusters were distinguished–*B*. *pumilus* (C2), *B*. *subtilis* (D), and *B*. *cereus* (H2) group. Moreover, unidentified isolates H19 and H28 were also placed close to the related strains which were corresponding species according to 16S rDNA identification–*B*. *licheniformis* (cluster D1) and *B*. *nealsonii* (G), respectively. MALDI-TOF MS analysis did not allow a reliable identification for isolates H19, H28, and H34, nevertheless, only the last two were identified at species level using molecular technique—as *B*. *nealsonii* and *B*. *limnophilus*, respectively.

### Impact of physicochemical properties of honeys on the bacterial composition

**Fig 4:**
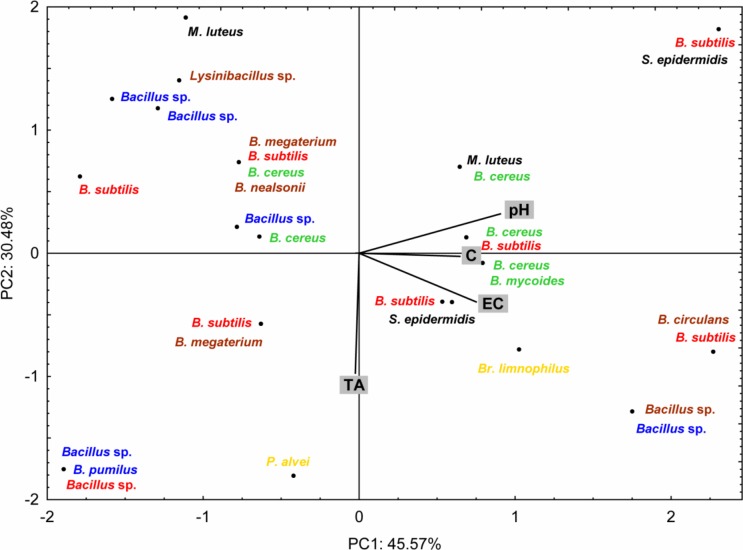
Influence of the physicochemical properties of investigated honeys on the bacterial species occurence (principal component analysis—PCA). **Bacterial names based on the both 16S rDNA sequencing and MALDI Biotyper identification.** C–color in Pfund scale; EC–electrical conductivity; TA–total acidity. Red—*B*. *subtilis* group; blue–*B*. *pumilus* group; green–*B*. *cereus group*; brown–other *Bacilliaceae*; yellow–*Peanibaciliaceae*; black–other species.

Grouping of the samples on the first two PCs-plane (76.05% of the explained variance) revealed that pH, EC as well as TA significantly influence the bacterial species composition of investigated honeys (correlation with the respective factors ≥ ±0.75) (Fig 4). Members of *B*. *cereus* group were most frequently present in the honeys with higher pH values (more alkaline), lower acids content as well as higher electrical conductivity. Contrary, isolates classified to the *B*. *pumilus* group preferred lower pH and were not affected by high acid content. The most ubiquitous type of bacteria among the analysed samples was *B*. *subtilis* group, for which occurrence was not significantly affected by the investigated physicochemical parameters. Regarding other types of bacteria, the residual representatives of *Bacilliaceae* family were more often present in the honeys with lower EC and pH values while *M*. *luteus* preferred low total acids content.

Considering botanical origin, the highest species diversity was observered in sunflower honeys– 5 different *Bacillus* species: *subtilis*, *pumilus*, *cereus*, *nealsonii*, and *megaterium*. The same number of species was demonstrated by samples of multiflorous and goldenrod honeys, however, representing less number of subgroups—4 and 3, respectively. Moreover, in the case of multiflorous samples, *B*. *cereus* was most dominant species– 5 out of 9 identified isolates. The lowest biodiversity among samples with multiple isolates was indicated for buckwheat honeys– 3 species, mostly *B*. *subtilis* (5 out of 7). Regarding geographical origin, most of the bacterial strains isolated from honeys derived from outside Poland belong to the *B*. *cereus* group– 5 compared to the 3 from polish ones, which constituted 56% and 10% of all isolates for each variant, respectively.

## Discussion

Results of our studies are in agreement with the generally accepted statement that Gram (+) bacteria are most often expected to be the highly dominant group [24] since due to high osmotic pressure honey is considered a harsh environment for the growth of microorganisms. Lack of the Gram (-) bacteria presence in investigated samples may indicate the application of good manufacturing practices during honey handling since their occurrence is most often associated with secondary sources of contamination such as people or equipment [10]. Moreover, 89% of the identified strains were able to produce endospores which indicate that their were rather present in a dormant form than in vegetative ones. Olaitan et colleagues [4] claimed that most of the microbial species cannot grow and reproduce in honey, thus, only spore-forming microorganisms can survive such conditions. Nevertheless, in 4 honeys presence of non sporulating *Staphylococcus epidermidis* and *Micrococcus luteus* was noted (Fig 1). As they are a part of the natural microflora of the human skin, they occurrence may be associated with handling and storage of honey without appropriate care on good manufacturing practices [12], [14]. On the other hand, *Micrococcus* spp. are typical colonizers of the hive and both *Micrococcus luteus* and *Staphylococcus* spp. have been recorded for gastrointestinal tracts of honeybees, from where they can be easily transferred to the honey [25]. For this reason, it is difficult to definitely assess whether the source of these strains of bacteria is primary or secondary. Nevertheless, it is emphasized that improved procedures of honey harvesting and handling is needed to reduce the introduction of such microbes as *Staphylococcus* species [24].

The vast majority of the isolated strains belonged to the genus *Bacillus* which should be expected since their symbiotic relationship with honeybees had been previously reported [26], [27]. Among them, two species (species groups)–*B*. *subtilis* and *B*. *cereus*—were the most frequently isolated strains (over half of all identified bacteria). Both species are ubiquitous in the environment and are regularly found in the honey [28], [29]. According to the literature, the presence of both mentioned species is associated with the potential spoilage of honey [19], however, *B*. *cereus* is the microorganism of particular concern since is classified as medically important human pathogen, emerging infectious agent, and principal foodborne pathogen at the same time [30]. Therefore, *B*. *cereus* presence in the honey should be under strict control. It does not change the fact that this species is regularly found in honey samples [27]. Iurlina and Fritz [8] reported occurrence of *B*. *cereus* in 23% among 70 Argentinean honeys which demonstrated presence of bacterial growth. Similar percentage of *B*. *cereus* in Argentinean honeys reported Alippi [29]– 20% and in works Monetto et al. [31] percentage of *B*. *cereus* in Argentinean commercial honeys reached 78%. In turn, studies on honeys derived from Turkey and Portugal revealed lower results– 4% and 14%, respectively [16], [3]. This findings may indicate the dependence of *B*. *cereus* presence on geographical origin of honey which is in agreement with results of our studies–the percentage of *B*. *cereus* among Poland honey samples was more than 4 times lower compare to the samples derived from abroad. Moreover, regardless of country of origin, most of the identified *B*. *cereus* were found in the multifloral honeys (5 out of 9). Considering the fact, that multifloral honey is the most common honey available in large quantities on the market [10], our and cited works may suggest to take special care of such kind of honey in terms of *B*. *cereus* presence as a potential vehicle of infection and the route of foodborne outbreaks.

According to the main purpose of the study, we used two different bacteria identification methods. While using 16S rDNA sequencing for honey bacteria idenitifcation is common, currently MALDI-TOF MS analyzer is getting more popular. Despite the increasing use of MALDI in the identification of clinical bacteria, the identification of environmental species is still limited [32]. This is due to the fact that commercial databases used for the MALDI approach contain less environmental reference spectra, in comparison with BLAST type repositories used in identifying microorganisms with 16S rDNA sequence [33]. Therefore in our study, the *Brevibacillus limnophilus* strain was identified only using 16S rDNA approach (Table 2). Its MALDI identification has not been possible due to the lack of reference spectra in the MALDI Biotyper database. Since it is believed that microorganisms originated from environmental samples are more diverse which implicate difficulties in their identification [20], Kopcakova et colleagues [34] emphasized the need to expand the current reference spectra database to improve the identification power of MALDI-TOF MS techniques in terms of environmental bacteria. One of the approaches to increase the identification power of the MALDI-TOF MS on genus and species level in case of environmental bacterial strains technique is the construction of home (local) databases with the parallel use of molecular techniques (eg 16S rDNA) [35]. Despite the fact that MALDI reference spectrum databases are more limited compared to molecular ones, percentage of the correct species identification via MALDI Bioptyper platform was almost 3 times higher compared to the 16S rDNA sequencing. Such findings resulted from dominance of the 2 big bacteria clusters–*B*. *subtilis* and *B*. *cereus* group. The first one comprises of such species as *B*. *subtilis* subsp. *subtilis*, *B*. *amyloliquefaciens*, *B*. *licheniformis*, *B*. *atrophaeus*, *B*. *mojaviensis*, *B*. *vallismortis*, *B*. *subtilis* subsp. *spizizenii*, and *B*. *soronensis* [36], while *B*. *cereus* is a parent species of group containing *B*. *thuringensis*, *B*. *mycoides*, *B*. *pseudomucoides*, *B*. *weihenstephanensis*, and *B*. *anthracis* [37], [29]. Distinguishing species within this two groups is difficult due to their high genetic similarities [38] which causes analysis of 16S rDNA sequences insufficient in distinguishing individual species [39]. Indeed, despite the very high 16S rDNA sequence similarity (>99.5%), all individuals were classfied only as *B*. *subtilis* group or *B*. *cereus* complex since distance scores to the next closest species were <0.5% and according to recommended guidelines are insufficient to reliable species identification, so additional housekeeping gene sequencing is required [40]. In turn, application of MALDI Biotyper enabled reliable species identification within *B*. *subtilis* and *B*. *cereus* group in most cases except for 4 strains most similar to *B*. *subtilis* as well as strain *H19* which was most similar to the *B*. *licheniformis* and *B*. *soronensis* species (Table 2). However, Similar findings were noted in works Lasch et al. [41], [42] or Fernandez-No et al. [43] in which obtained accurate classification of *B*. *cereus* and *B*. *subtilis* group. In contrast, in the distinguishing of *B*. *licheniformis* and *B*. *soronensis* MALDI approach is considered as not very useful since their share more phenotypic traits with each other than with any other taxon [44]. In another study Dieckmann et al. [45] noted that 16S rDNA sequencing failed to resolve problems with intra- and inter-species classification of *Pseudoalteromonas* sp. isolates derived from marine sponges while application of MALDI-TOF MS technique enabled discrimination of very closely related species with high confidence. Similar findings revealed Angolini et al. [46] investigating petroleum microorganisms. It suggests that use of proteomic-based approaches such as MALDI-TOF MS is a good solution for distinguishing some species sharing very similar 16S rDNA sequences as in the case of honey bacteria, without the need to analyze additional genes. MALDI approach seems to be more efficient for *Bacillus sp*. identification e.g. distinction between *B*. *cereus* and *B*. *subtilis*, in comparison with 16S rDNA sequencing. However, MALDI has also some restrictions in *Bacillus cereus* group differentiation. As an example, proper identification beetwen *B*. *cereus* and *B*. *anthracis* is still challage [47].

Revealed differences in identification quality between *B*. *cereus* and *B*. *subtilis* group may results from influence of endospores production. It is known that proteins expression in endospores differ from vegetative cells of *Bacillus* species mainly due to high amounts of small acid soluble proteins which play crucial role in endospore formation [48]. It was observed, that with increasing time of incubation from 12-48h the numbers of spores increase, while the number of signals on the MS spectrum decreases. This causes a reduction of the identification power of MALDI approach. Shu and Yang [49] claimed that fresh cultures (<12h) are ideal samples for classification and identification of *Bacillus* species since inconsistencies of their identification are mostly because of endospores production which is dramatically influenced by incubation time. Based on UK standards for Microbiology Investigations Identification of Bacillus species, *Bacillus cereus* and *Bacillus subtillis* belongs to two different endospores groups with different time of sporulation which may influence quality of MS spectra and thus further bacteria identification. As a solution for this problem Lasch et al. [42] proposed the protein enrichment protocol where application of combined TFA treatment promoted the protein isolation from spores. However, this analytical solution does not prevent the sample from isolation of protein from mixed bacterial culture (different time and stage of sporulation), on the one hand, leading to an increase of proteins signals in MALDI spectra, but on the other hand causing misidentification [50]. However, there are some reports which suggest that in the case of *Bacillus* strains, species identification could be also performed based only on the protein profiling of their spores using both top-down and bottom-up approaches which was proved for such species as *B*. *cereus*, *B*. *globigii* or *B*. *atrophaeus* [51], [52]. Nevertheless, considering vegetative cells, our results suggest using different MALDI sample pre-treatment protocols for individual group of honey bacteria contradicts the statement of Santos et al. [20] that there is the need of a universal sample pre-treatment protocol to overcome misidentification problems.

Results of our studies also revealed that both physicochemical parameters, as well as the origin of the honey, have a significant influence on the bacterial composition since they are very often correlated with each other. However, this effect strongly depended on the bacterial species. Occurrence of the *B*. *cereus* strains was considerably influenced by the TA, pH as well as EC values, while, in the case of *B*. *subtilis* no significant differences were observed (Fig 4). *B*. *cereus* strains mostly preferred more alkaline honey, thus with lower acid content. The impact of EC, which in honey is associated with ash content, was lower, however, still statistically significant. It was found that among the most important variables associated to the levels of bacteria in honey were ash (related to the solids and mineral content) and acidity [53]. It is also believed that in undiluted honey the acidity is a significant antibacterial factor [4] since the optimum pH for most bacteria is between 7.2 and 7.4 while natural acidity of the honey ranges between pH 3.2 and 4.5 [54]. It is believed that among *Bacillus* spp. *B*. *cereus* generally reflects higher tolerance to antimicrobial properties of the honey[19]. The results of our research are contradictory to this statement because *B*. *cereus* showed the least tolerance among all identified *Bacillus* species. Nevertheless, impact of the botanical origin of honey must be also taken into account since is associated with some physicochemical properties such as color and thus mineral and organic content including acids (pH) [4], [55]. Sinacori et colleagues [5] investigating various types of honeys have shown that the microbiota of multifloral honeys showed the highest values for genotype richness and diversity indexes. Moreover, the authors revealed that some species were detected in almost all honeys, which may indicate their strong adaptation to such kind of matrix, while some species were detected only occasionally, and thus their presence cannot be correlated with the honey origin. Similar to this findings, investigated samples of multiflorous honeys found themselves in a group of honeys with the highest species diversity of bacteria with dominance of *B*. *cereus* strains, while strains belonged to the *B*. *subtilis* group proved to be the most ubiquitous.

## Conclusions

Our studies revealed that regardless geographical and botanical origin, honeys are by far dominated by spore-forming *Bacillus* spp. According to literature and our own observations, the most frequently isolated honey bacteria belong to the *B*. *subtilis* or *B*. *cereus* group, which raises big problems with identification to the species level via 16S rDNA technique. Solution for this problem in some cases can be using MALDI-TOF MS and suitable software, such as MALDI Biotyper platform. Application of such an approach can significantly improve quality of the identification analysis–in our studies nearly three times–and avoids the need for sequencing of additional housekeeping genes so lets keep short laboratory workflow. It is particularly important in the case of *B*. *cereus*, which is a microorganism of special concern in terms of honey spoilage and foodborne illness outbreaks. However, the influence of endospores formation should be taken into account since it significantly affects MS profiles and thereby microbial identification. As a solution for this obstacles, shorter incubation times (less than 12 hours) are recommended. Nevertheless, in the case of *B*. *cereus* group even the use of a standard 24-hour incubation protocol seemed to be sufficient for reliable species indentification. Considering that both the geographical and botanical origin as well as the physicochemical parameters of honeys, affecting the composition of bacteria and the fact that these parameters are very often related, this indicates a great need for further studies on a larger scale using a greater number of different types of honey. For this purpose use of the MALDI-TOF MS technique seems to be the most promising approach, as indicated by our study. Such studies can significantly expand knowledge about microbial composition and occurrence of the specific species in the different type of honey which are relevant in view of safe food handling and processing. This might give the opportunity to consider honey as a source of microorganisms which are able able to survive in suboptimal conditions (e.g. high sugar concentrations) or as good material for microbial inocula preparation.

## Materials & methods

### Honey samples

During the study, 20 different honeys belonging to the collection of the Department of Environmental Chemistry and Bioanalytics of the Nicolaus Copernicus University in Toruń were tested. Most of them– 14 –derived from Poland while the rest originated from different countries around the world–Australia (3), Italy (1), Ukraine (1), and Portugal (1). Moreover, honeys varied in botanical origin: multiflorous– 4; buckwheat– 3; honeydew, rape, sunflower, and goldenrod– 2; clover, leatherwood, bush, forest as well as lime– 1. Full names of the honeys with given acronyms are presented in Table 1.

### Colour of honey samples

Collected honey samples (4 g of each, dissolved in water 8 mL) were heated up to 50°C to dissolve sugar crystals. The spectrophotometric measurements were performed by use of UV-Vis spectrophotometer NanoDrop 2000c (*Thermo Fisher Scientific*, Waltham, MA USA). The colour of sample was determined by measurement of the absorbance of a 50% honey solution (w/v) at wave length λ = 635 nm. The honeys were classified according to the Pfund's scale after conversion of the absorbance (Abs) values:

mmPfund=−38.70+371.39×Abs

where mm Pfund is the intensity of honey colour in the Pfund's scale; Abs is the absorption of honey solution. Two replicates were performed for each honey sample.

### pH and acidity of honey

10 g of honey was dissolved in water 75 mL. Next pH of the solution was measure by use of pH-meter CPC-501 (*Elmetron*, Chorzów, Poland). A glass electrode was used. Measurements have been made in triplicate.

After measurements of pH, in a beaker with a honey solution a magnetic stirrer was placed and the titration continued by use of standard solution NaOH (0.1 M) to obtain pH 8.3.

$$Total\;acidity\;(TA)\;[mval/kg]=V_{NaOH}\times 10$$

V_NaOH_−volume of NaOH 0.1 M solution used during the titration of honey solution

### Conductivity of honey

The electrical conductivity (EC, mS × cm^–1^) of the honey has been measured for 1 mL of water solution (20%) of the honey, on a dry matter basis, at the temperature 20°C. At first 2 g of honey was dissolved in water 8 mL. The sample was transferred to the conductivity cell and the conductivity of the solution was measured by use of equipment CPC-501 (*Elmetron*, Chorzów, Poland).

Measurements were performed for each sample in the triplicate repetition. Data are presented as mean values. The level of statistical significance required to measure differences between the means for all analyses was P = 0.05.

### Bacteria isolation

For isolation of aerobic bacteria, the serial dilution method was used. 10 g of honey was added into the 90 ml of sterile physiological saline solution (0.87% NaCl), mixed well, and then 100 μl of obtaining suspension (10^−1^) wereplated on TSA medium (Triptic Soy Agar, Sigma-Aldrich, Germany) and incubated for 24h at 37°C. After incubation, single colonies were transferred onto new TSA plates in order to obtain pure bacterial cultures using reductive culture method. Pure cultures were stored on TSA slants at 4°C. Microorganisms from the same passage were used for both identification methods.

### 16S rDNA identification of bacterial isolates

Total bacterial DNA was extracted from overnight cultures (TSA, 37°C) using the Bacterial Genomic Extraction GPB Mini Kit and GPB Lysozyme (GenoPlast Biochemicals, Poland). Regions of 16S rDNA were amplified using universal primers for bacteria: 27F (5-AGAGTTTGATCMTGGCTCAG-3) and 1492R (5-GGTTACCTTGTTACGACTT-3), thermostable Taq DNA polymerase (Qiagen, Hilden, Germany), Mastercycler pro S thermocycler (Eppendorf AG, Hamburg, Germany), and following PCR program: 1. 95°C, 1 min. (initialization); 2. 95°C, 15 s (denaturation); 3. 55°C, 15 s (annealing); 4. 72°C, 90 s (elongation): 2.– 4. repeated 30x; 5. 72°C, 7 min (final elongation); 6. 10°C (cooling for final hold). PCR products were then purified using Extractme Genomic DNA kit (Blirt S.A., Poland) followed by direct DNA sequencing via sanger dideoxy method using the same 27F and 1492R primers. Quality of obtained chromatograms of 16S rDNA sequences were checked using Chromas ver. 2.6.2 software (Technelysium Pty Ltd, Australia). Contigs were assembled via BioEdit Sequences Alignment Editor ver. 7.2.5 (Tom Hall, USA), and finally, consensus sequences were compared with known 16S rDNA genes present in the The National Center for Biotechnology Information (NCBI) BLAST database [56]. Obtained sequences were submitted to GenBank and received accession numbers. Evolutionary relationships of investigated bacterial strains were presented on a phylogenetic tree created using the Neighbor-Joining method [57] with computing the evolutionary distances by the Maximum Composite Likelihood method (bootstrap test—500 replicates, values lower than 70% were hidden) [58] via MEGA7 ver. 7.0.21 software [59].

### MALDI-TOF/MS identification of bacterial isolates

α-Cyano-4-hydroxycinnamic acid (HCCA) solution in standard solvent (acetonitrile (ACN) 50%, water 47.5% and trifluoroacetic acid 2.5%) at final concentration: 10 mg/ml was used as matrix. For sample preparation, ethanol/formic acid (EtOH/FA) extraction procedure was performed according to Bruker’s guideline with small modifications. A single colony of bacteria was transferred into an Eppendorf tube containing 300 μl of deionizated water and mixed thoroughly. Subsequently, 900 μl of absolute EtOH were added and thoroughly vortexed, then centrifuged at max speed for 20 min. The supernatant was discarded, and the remaining cell pellet was dried by evaporation of EtOH residue at 37°C for 5–10 minutes to increase the extraction efficiency. Then, 1–5 μl of 70% FA was added to the dried cell pellet proportionally to the amount of biological material and mixed by pipetting. Next, an equal volume of can was added, mixed carefully, centrifuged at max speed for 3 min., and 1 μl of supernatant was transferred onto a MALDI MTP 384 ground steel target sample spot (Bruker Daltonik GmbH, Germany). Finally, the dried sample spot (after ~15 min.) was overlaid with 1 μl of HCCA matrix solution and air dried. Target with samples was analyzed in an ultrafleXtreme MALDI–TOF/TOF mass spectrometer (Bruker Daltonik GmbH, Germany) equipped with the smartbeam-II laser–positive mode. The cut-off for applied liner MBT Standard methods (Bruker Daltonik GmbH, Germany) was at *m/z* range: 2000–20 000, acceleration voltage at 25 kV, global attenuator offset at 20% and attenuator offset at 34% and its range at 34%. Applied laser power at 40% and smartbeam parameter set at MBT focus were applied. The one single spectra above 8000 unit intensity was collated manually by 500 shots in-one-single spectra to frequency 2500. For calibration Bruker Bacterial Test Standard (BTS), algorithm Reference Mass Assignment and interactive calibration with of quadratic mode. Before calibration, each spectra of bacteria were subjected to smooth and baseline operations. Validated mass spectra were processed with the use of software provided by the manufacturer–flexControl and flexAnalysis, and subsequently used for bacterial identification via MALDI Biotyper Compass platform (Bruker Daltonik GmbH, Germany) based on the both raw spectra (RAW) and Main Spectra (MSP) according to manufacturer protocol [60]. The spectra of 3 microbial replicates was collected in triplicate.

### Statistical analysis

Effect of investigated physicochemical properties of honeys on their bacterial composition was analysed by principal component analysis (PCA) using STATISTICA version 12 software (StatSoft, Poland). During the analysis, the results of 16S rDNA identification and covariance as a measure of the linear relationship between variables were used.
